# RETRACTION: “The Morphological Features and Mitochondrial Oxidative Stress Mechanism of the Retinal Neurons Apoptosis in Early Diabetic Rats”

**DOI:** 10.1155/jdr/9790267

**Published:** 2025-11-13

RETRACTION: X. Li, M. Zhang, and H. Zhou, “The Morphological Features and Mitochondrial Oxidative Stress Mechanism of the Retinal Neurons Apoptosis in Early Diabetic Rats,” *Journal of Diabetes Research,* no. 2014 (2014). https://doi.org/10.1155/2014/678123.

The above article, published online on 2 January 2014 in Wiley Online Library (http://wileyonlinelibrary.com), has been retracted following the agreement of the journal's Associate Editor, Bright Starling Emerald; and John Wiley & Sons Ltd.

The retraction has been agreed following an investigation of the concerns raised by *Penicillium citreoviride* and *Actinopolyspora biskrensis* on PubPeer [1], which identified concerns regarding Figures 2 and 3.

Specifically, the panels “e” and “m” of Figure 2 share overlapping features, despite showing retinal structure of animals in different treatment groups. Moreover, the panels “c” and “d” of Figure 3 show the same tissue, however they are attributed to different treatment groups.

As a result of the investigation, the data and conclusions of this article are considered unreliable.

The authors have been informed of this retraction but did not provide a response.
